# Computer-Assisted School-Based Asthma Management: A Pilot Study

**DOI:** 10.2196/resprot.1958

**Published:** 2012-11-13

**Authors:** Renée JG Arnold, Jeanette A Stingone, Luz Claudio

**Affiliations:** 1 Mount Sinai School of MedicinePreventive MedicineNew York, NYUnited States; 2 Arnold Consultancy & Technology LLCNew York, NYUnited States; 3 University of North Carolina, Chapel HillGillings School of Global Public HealthDepartment of Epidemiology, CB#7435Chapel Hill, NCUnited States; 4 Mount Sinai School of MedicineOne Gustave Levy PlaceNew York, NYUnited States

**Keywords:** Asthma, disease management, Internet, child, underserved, Asthma Action Plan, outcomes, urban, low-income

## Abstract

**Background:**

The high prevalence of asthma among children continues to be a major public health issue. In particular, low-income African-American and Hispanic children often receive asthma care in the emergency department and lack access to continuity of care.

**Objective:**

The aim of the current study was to test the feasibility of implementing a computerized program for empowering low-income children with asthma to manage their own disease. This pilot program consisted of a guided, personalized, Web-based computer program as the main component of a school-based asthma intervention.

**Methods:**

The Automated Live E-Health Response Tracking System (ALERTS), a computer-assisted, Web-based tracking program, was tested for implementation in a school in East Harlem, New York. The program required children with asthma, assisted by trained researchers, to routinely measure their peak flow meter readings and answer a symptom questionnaire. The program provided individualized feedback on their disease status based on peak flow meter input. The computer program sent reports to the child’s physician and the nurse practitioner at the on-site school health center. The children were also encouraged to bring the reports home to their parents. A pre/post study design was employed such that each participant acted as his/her own control. Comparisons of preintervention and postintervention outcomes were calculated using the paired *t*-test and the McNemar test for dichotomous data.

**Results:**

Twenty-four children (6 to 12 years) participated in the program over 2 to 15 months. Improvements in health outcomes showed the greatest significance among the group of participants who were enrolled for 8 months or longer. Statistically significant improvements were seen in the average physical health score of the children (from 65.64 preintervention to 76.28 postintervention, *P* = .045). There was a significant decrease in the number of participants experiencing wheezing episodes (n = 9 to n = 2, *P* = .03), and in the average number of wheezing episodes per child (1.86 to 0.43, *P* = .02). Although not statistically significant, decreases were also seen in the number of children experiencing an asthma attack and in the average number of asthma attacks among participants. There was also a significant decrease in the average number of visits to doctors’ offices or clinics (1.23 to 0.38, *P* = .04). There were no overnight hospitalizations in the two-week period following the end of the pilot program, a nonsignificant reduction from an average of 0.21 per child.

**Conclusion:**

This individualized, computer-assisted intervention resulted in improvements in some health outcomes among low-income children in an urban, public school-based setting. Consistent peak flow meter self-measurements, management of medication usage, and a computerized approach to symptom tracking resulted in fewer asthma exacerbations and improved overall physical health among this pediatric population with asthma.

## Introduction

The high prevalence of asthma among children continues to be a major public health issue in the United States and it has both social and economic impacts [1-4]. East Harlem is a low-income, predominantly African-American and Hispanic community in New York City with one of the highest rates of asthma hospitalizations in the United States, measured at 27.6% in 2006 [5-7]. Previous research has shown that people living in low-income urban settings, such as those living in East Harlem, often receive asthma care in the emergency department, have the highest rates of hospital admission, lack access to follow-up medical care, do not receive education about their disease, and lack information to improve their self-management skills [7-13].

Researchers have determined that in order to improve health outcomes it is key to ensure that patients are educated about self-management of asthma and asthma attacks [14]. One potential pathway to reducing the burden of asthma in children is through the use of school-based programs. Children spend the majority of their day at school and are accustomed to receiving health education in this setting. Although Oruwariye et al [15] determined that school-based health centers often provided inadequate asthma care, other studies have concluded that health centers within elementary schools can produce a reduction in the rate of hospitalization and a slight decrease in absenteeism in children with asthma [16]. Moreover, school-based asthma programs have been shown to be effective regardless of whether they have a school-based health center [17-23]. Nonetheless, targeted interventions implemented in conjunction with school-based health centers could take advantage of existing health-based infrastructures while providing a personalized level of asthma education and care that could help increase children’s self-management skills. A meta-analysis examining 32 educational interventions for self-management of asthma in children concluded that programs that promote self-management in the medical care of children with asthma may greatly improve their health outcomes. The analysis also showed that programs that incorporated peak flow measurements as a main component of the intervention and programs that were individually tailored to the patient (vs addressed to groups) showed the greatest success in reducing disease morbidity [24].

Some interventions in urban communities, such as East Harlem, have been implemented in conjunction with school-based health clinics. Results have been mixed, sometimes even within the same study, with some studies showing improvement in health outcomes, morbidity, and health care utilization (eg, fewer hospitalizations, emergency department visits, and follow-up visits for asthma) [25-29]. Some of the same studies have also shown positive changes in levels of asthma knowledge, self-management skills, and use of peak flow meters and inhalers, but little to no improvement in clinical outcomes [26,28,29].

The use of computers as a method to improve asthma self-management skills is a relatively new technique. It has proven successful in other studies helping children adhere to prescribed medication plans and in reducing the number of asthma exacerbations they experience, although not all of these interventions were school-based [28,30-35]. Computer-assisted asthma management programs have the potential to provide children with individualized, evidence-based feedback on their disease, including measurements of symptom severity and frequency, and can provide actual data on medication usage rather than leaving children and parents to rely on perceived or recalled medication usage information [30, 36]. We implemented a computer-assisted, Web-based, tracking pilot program that required children with asthma to routinely provide their peak flow meter readings. The program provided individualized, tailored feedback on their disease status based on this input.

### Aim

The aim of the current study was to test the feasibility of implementing a computerized program for empowering children with asthma living in a low-income, urban setting to manage their own disease. This pilot study used a guided, personalized, Web-based computer program as the main component of a school-based asthma intervention.

## Methods

### Participants

The computer-assisted asthma management pilot program was implemented in a public elementary school in East Harlem, New York. At the time the program began, the school had enrollment of 588 students (49.1% Hispanic and 46.6% African American) in prekindergarten through grade 6. More than 95% of students qualified for the free lunch program, a surrogate measure of low socioeconomic status [37]. The school houses a health center that operates during the day, evening, and on weekends for 12 months per year to provide comprehensive community health services, including routine preventive care, chronic disease management, and treatment for minor illnesses.

### Enrollment Process

The study was reviewed and approved by the Mount Sinai School of Medicine Institutional Review Board, the Mount Sinai Health Insurance Portability and Accountability Act (HIPAA) Privacy Officer, and the New York City Department of Education’s Institutional Review Board. Prior to implementing the intervention, a needs assessment was conducted in the school to quantify the magnitude of the asthma problem and to identify possible gaps in asthma management plans. A questionnaire that contained standardized items on demographics, indoor environmental factors, asthma diagnosis and symptoms, and the use and access to medical care and medications for asthma was developed and distributed. The questionnaire also included items on whether children had an asthma management plan and what details were included in that plan. Questionnaires were provided in English and Spanish. Children were instructed on the importance of their participation and were asked to bring the questionnaires home to be completed by their parents or guardians. Teachers were also encouraged to maximize class response rates; classrooms with over 80% response rates were given gift certificates for school supplies.

The needs assessment had an overall response rate of 68% and identified 101 students within the school who had active asthma (defined as a physician’s diagnosis of asthma and asthma symptoms in the previous 12 months). Among these children with active asthma, over 19% did not have an asthma management plan. Most (69.3%) of the parents of children with active asthma had not been given information on when to call the doctor to assist with their child’s asthma. Most of the children with asthma (59.4%) had been treated at an emergency department for their asthma in the previous 12 months. Parents of children with asthma were contacted via telephone and invited to meet with the study coordinators in a face-to-face meeting at the school to discuss their child’s potential participation in the study. If a parent voluntarily agreed to the child’s participation, informed consent was obtained at this time, in either English or Spanish. A total of 24 children with asthma were enrolled in the pilot study.

Once students were enrolled in the program, their asthma control was classified according to the National Heart, Lung, and Blood Institute (NHLBI) guidelines using the patient/parent responses to a baseline questionnaire. The control level of the child’s asthma determined how often the child would access the program each week. Students with “mild intermittent” or “mild persistent” asthma accessed the program once per week, whereas those classified as having “moderate persistent” asthma used the program three times per week. Participants with “severe” asthma were required to use the program daily. A pre/post study design was employed, such that each participant acted as his/her own control.

### Symptom Questionnaire

After inputting peak flow meter readings into the computer program, students were prompted to answer a symptom questionnaire in addition to questions about medication usage and physician and emergency department visits that occurred since their previous input. The symptom assessment questionnaire included questions regarding the frequency of symptoms (ie, shortness of breath, cough, wheezing, tightness in the chest, difficulty sleeping, and limitations to physical activity). The children were also asked to complete weekly event questionnaires that included questions about the presence or absence of a persistent cold, frequency of use of a fast-acting (rescue) inhaler, use of inhaled and oral steroids, days of school missed, and visits to the physician, emergency department, or hospital. Medication use was assessed by showing pictures of various asthma medications and prompting children to point to the one they used.

### Outcomes Assessment and Data Analysis

To evaluate the effectiveness of the intervention, parents of the student participants were asked to complete the Children’s Health Survey for Asthma (CHSA) at two different time points: (1) before the program began, and (2) up to 3 weeks after their child’s final day of participation in the program [38]. The program was implemented over the course of 2 school years, constituting 2 phases of enrollment. Parents were asked to complete a CHSA either at the end of each school year or at the end of their child’s enrollment in the program, whichever came first. Parents were given a US $20 incentive for completing the questionnaire. Children did not complete the CHSA.

The CHSA, developed and validated by the American Academy of Pediatrics, is a validated assessment questionnaire that contains questions about asthma symptoms, health care utilization, quality of life, physical activity, and family support; it has been used in numerous studies of pediatric asthma [38-40]. Numeric answers to questions in a Likert-scale format are compiled to create composite scales, ranging from 0-100, with higher scores indicating better outcomes. The five scales that can be computed from the questionnaire measure the physical health of the child, the activity level of the child, the activity level of the child’s family, the emotional health of the child, and the emotional health of the child’s family. The core items used to compute the physical health score are questions about symptoms due to asthma, such as tightness in the chest and wheezing, and symptoms due to use of asthma medicines, such as rapid heart rate and irritability. The core items used to compute the activity level scores are questions about how limited children were from participating in various activities, such as gym class and playing or running outside. Respondents were asked to answer questions about their children’s health based on symptoms, health care utilization, and other outcomes that occurred during the 2 weeks prior to the day that respondents completed the questionnaire.

Outcomes data were analyzed using SPSS version 12 (SPSS Inc, Chicago, IL, USA). Comparisons of preintervention and postintervention outcomes were calculated using the paired *t*-test and the McNemar test for dichotomous data.

### Intervention

**Figure 1:**
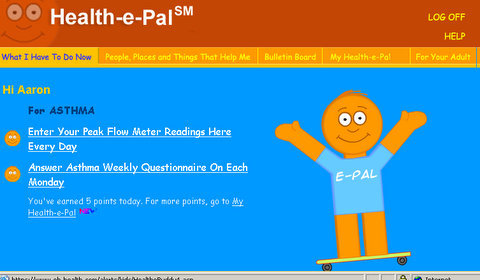
Pediatric interface of Health-e-Pal ALERTS program.

The Automated Live E-Health Response Tracking System (ALERTS) was developed as a computer-assisted, Web-based, symptom-tracking program that allows patients with chronic disease to monitor their symptoms while reinforcing key educational lessons. Although the program was available through both a toll-free phone number and the Internet, children accessed the program only via the Internet in this study. The web-based display showed a pediatric or adult patient interface, depending on the user’s profile (age ≤ 18 years displayed a pediatric interface). Researchers trained each student individually on how to use the computer program. First, the researchers demonstrated the correct way to use a peak flow meter and then coached the children on their own technique. Once the children had learned the proper technique for peak flow measurement, the researchers instructed them on the use of the computer program. Users were issued a unique personal identification number (PIN) for confidentiality and used this PIN to log in to each session. A room within the school was designated to house the computers where children accessed the program and children were coached to use the program during their lunchtime. The computer program interface is shown in Figure 1.

In their first session, the researchers showed the children how to enter their unique PIN and password into the interface in order to access the program and then they supervised the children as they repeated the process. Once the program interface was loaded, children were encouraged to read the screens aloud and ask the researchers any questions as they completed the program tasks and answered symptom questions. In each subsequent session, students independently logged in to their saved information settings using their individual PINs, and then inputted their current peak flow meter readings under the supervision of research staff. Children were escorted from the cafeteria to measure their peak flow meter readings during their lunch periods. To maintain consistency in the readings, the program provided an input box to be checked to indicate whether the peak flow meter reading was taken in the morning (before noon) or afternoon.

Based on the daily peak flow meter input, the program provided evidence-based feedback regarding each child’s symptom zone. Specifically, when peak flow meter readings were within 80% of the child’s personal best peak flow meter reading, the program let the child know they were in the “green zone;” if within 50%-80% of the child’s best, they were in the “yellow zone;” and when below 50%, they were in the “red zone.” The program then instructed children on how to adjust their medication use or modify activities according to their prescribed Asthma Action Plans. If a child’s peak flow meter reading was not in the green zone, a report was automatically generated and brought by the child to the school health center and to research staff in the nurse’s office. If a child’s peak flow meter reading was in the red zone, a member of the research team escorted the child to the school health center. Children were given small toys, pencils, stickers, and other school supplies as incentives for their participation in the program.

**Figure 2:**
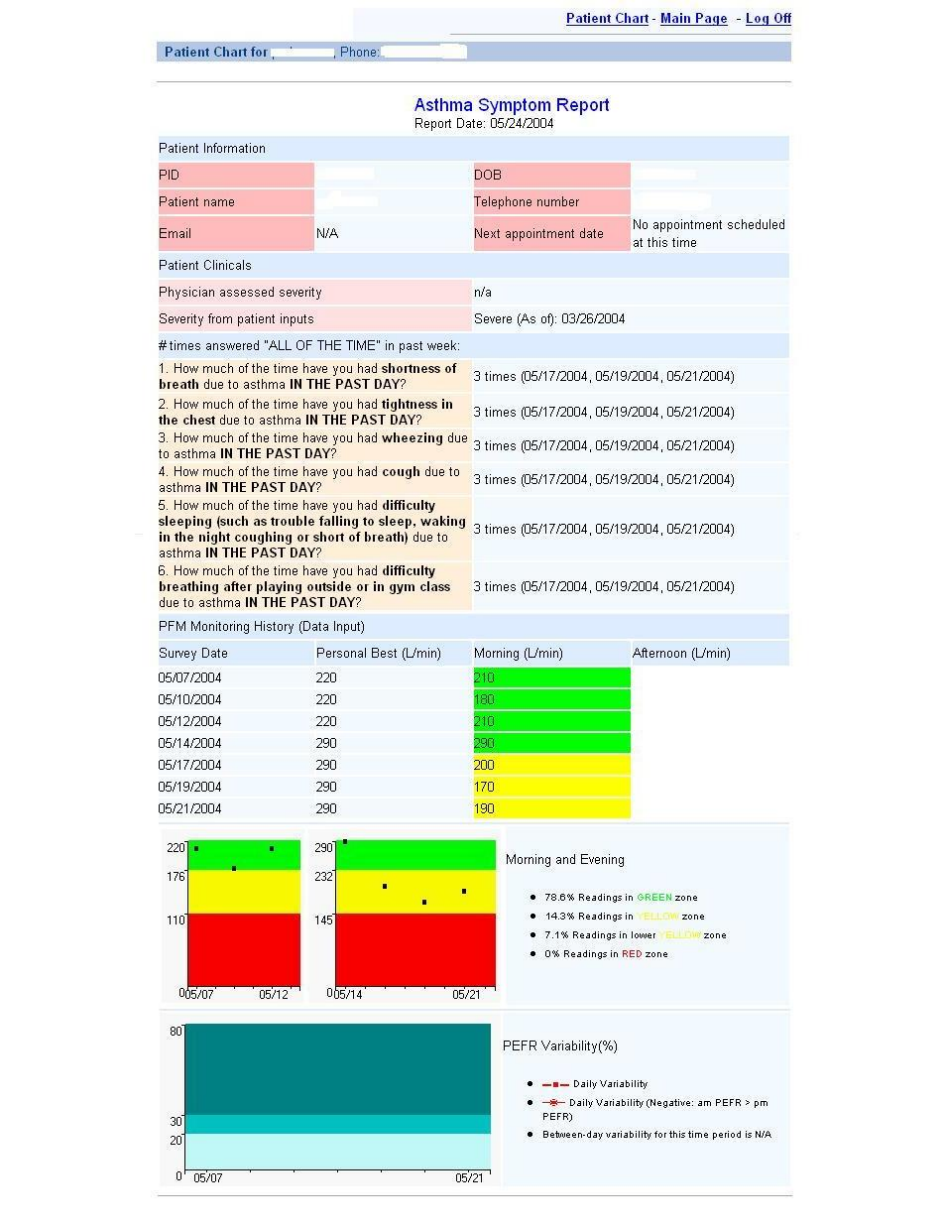
Asthma Intervention Report (AIR) Symptom Report.

The system was designed to send reports to the child’s physician and the nurse practitioner in the on-site school-based health center. The provider received two types of reports based on questionnaires the children completed: (1) the Asthma Intervention Report (AIR) Symptom Report (Figure 2), which tabulated patients’ self-reported peak flow meter readings and affiliated symptom reporting, and (2) the AIR Event Report (not shown), which tracked weekly events related to asthma, such as urgent visits to physicians or clinics and hospitalizations. Children were given real-time, evidenced-based feedback regarding their symptom zone and what to do according to their prescribed Asthma Action Plan. Program staff showed the children their peak flow meter history and symptom reports periodically and the children were encouraged to review how their peak flow meter readings had changed over time. Children were asked to identify if their readings had been increasing, decreasing, or staying the same. At the end of the program, paper copies of the peak flow meter history and symptom history were given to the parents of all participating children to share with their child’s health care provider.

## Results

### Results of Needs Assessment and Population Demographics

Of the 588 students enrolled in the school, 389 returned the asthma prevalence questionnaires, a response rate of 66.2%. Half (50.9%, 198/389) of respondents identified themselves as Hispanic, 36.0% (140/389) as black or African American, 1.5% (6/389) as white, 0.7% (3/389) as Asian, 3.9% (15/389) as “other,” and 6.9% (27/389) did not give an answer. The average age of students surveyed was 8.2 years. Of all responding students, 57.6% (224/389) were living in a household with a yearly gross income of less than US $20,000. A total of 38.0% (148/389) of all students had been diagnosed with asthma at some point in their lives and 26.0% (101/389) had current asthma (defined as a diagnosis with symptoms within the previous 12 months) and were identified as “current patients with asthma” (Table 1).

**Table 1:** Characteristics of students who completed the needs assessment questionnaires.

Characteristics		Respondents, n (%)	
		All students n = 389	Children with active asthma n= 101
**Sex**			
	Male	186 (47.8%)	59 (58.4%)
	Female	190 (48.8%)	38 (37.6%)
	No answer given	13 (3.3%)	4 (4.0%)
**Race**			
	Hispanic	198 (50.9%)	52 (51.5%)
	Black or African American	140 (36.0%)	41 (40.6%)
	White	6 (1.5%)	0 (0.0%)
	Asian	3 (0.7%)	0 (0.0%)
	Other	15 (3.9%)	4 (4.0%)
	No answer given	27 (6.9%)	4 (4.0%)
**Household gross income (US $)**			
	Less than $20,000	224 (57.6%)	67 (66.3%)
	$20,001-$39,999	95 (24.4%)	19 (18.8%)
	$40,000-$74,999	23 (5.9%)	5 (5.0%)
	Over $75,000	3 (0.8%)	0 (0.0%)
	No answer given	44 (11.3%)	10 (9.9%)
Students ever diagnosed with asthma		148 (38.0%)	--
Students diagnosed with current asthma		101 (26.0%)	--
Emergency department visits due to asthma in previous 12 months		N/A	60 (59.4%)
Hospitalizations due to asthma in previous 12 months		N/A	16 (15.8%)
Have a peak flow meter		N/A	36 (35.6%)
Use a peak flow meter daily		N/A	31 (30.7%)
Have a spacer		N/A	47 (46.5%)
Use a spacer regularly		N/A	26 (25.7%)

Of the 101 students identified as having current asthma, 59.4% (60/101) had visited an emergency department for urgent care due to asthma in the previous 12 months. In addition, 15.8% (16/101) of all current patients with asthma had been hospitalized due to asthma in the previous 12 months. Our needs assessment showed that of all current asthmatics, 35.6% (36/101) owned peak flow meters but only 30.7% (31/101) of them used it daily. Similarly, although 46.5% (47/101) of current asthmatics had a spacer for use with inhaled medications, only 25.7% (26/101) said they used a spacer regularly.

### Computer-Assisted Intervention: Participant Demographics and Pretest and Posttest Results

#### Demographics

A total of 24 students were enrolled in the pilot program, ranging in age from 6-12 years at the time of enrollment. Of these, 63% (15/24) of the participants were male and 79% (19/24) were Hispanic. Average household income for 46% (11/24) of participants was less than US $14,999. Most of the children were classified as having mild/ intermittent asthma, as defined in NHLBI guidelines (See Table 2).

**Table 2:** Baseline demographic characteristics of program enrollees^a^(n = 24).

		n	%
Average age, mean (years)		8.6	--
Length of enrollment in program, median (months)		12	--
**Sex**			
	Male	15	63%
	Female	9	37%
**Race**			
	Black or African American	4	17%
	Hispanic	19	79%
	Other	1	4%
**Student household yearly gross income (US$)**			
	Less than $14,999	11	46%
	$15,000-$29,999	6	25%
	$30,000-$60,000	2	8%
	No answer	5	21%
**Severity of asthma, according to NHLBI guidelines**			
	Mild intermittent	15	63%
	Mild persistent	6	25%
	Moderate	2	8%
	Severe	1	4%

^a^Data from Children’s Health Survey for Asthma (CHSA).

The children participated in the pilot study for 2 to 15 months over the course of 2 school years. Median enrollment time was 12 months. Nine students (38%) remained in the pilot for 7 months or less and 15 students (62%) were enrolled for 8 months or more. (Enrollment time includes only the months that school was in session and excludes summer break months.) The asthma control levels of the participants varied, with 15 having mild intermittent asthma, 6 having mild persistent, 2 having moderate, and 1 having severe asthma. Participants who remained enrolled in the program for 8 months or more tended to have more severe asthma. In contrast, children with milder symptoms tended to stop participation in the study at an earlier point.

#### Children’s Health Survey for Asthma

Of the 24 student participants, 17 had a parent or guardian who completed the CHSA both at baseline and after completing the program. Four participants completed only one CHSA and 3 participants did not complete a CHSA and were excluded from further analysis. It is worth noting that the 3 enrollees who did not complete a CHSA were classified with the lowest level of asthma severity (mild intermittent), which may have contributed to their attrition from the program. Of the 17 participants who had paired (before and after intervention) CHSA results, improvements in health outcomes showed the greatest significance among the group of participants who were enrolled for 8 months or longer. A number of improvements in health outcomes were observed in our analysis (Table 3).

**Table 3:** Asthma outcomes among pilot program participants who completed preintervention and postintervention Children’s Health Survey for Asthma (CHSA) surveys (n = 14).

Outcome	Prepilot (n or mean)	Postpilot (n or mean)	*P* value
Average physical health score (child)	65.64	76.28	.045^a^
Average physical activity score (child)	75.18	83.57	.24
Average physical activity score (family)	81.85	90.83	.11
Average emotional health score (child)	64.64	77.14	.17
Average emotional health score (family)	68.60	68.79	.97
Participants with asthma attacks^b^, n	7	3	.13
Participants with wheezing^b^, n	9	2	.02^a^
Average number of asthma attacks^b^	1.43	0.50	.10
Average number of wheezing episodes^b^	1.86	0.43	.02^a^
Average number of visits to doctor or clinic^b^	1.23	0.38	.04^a^
Average number of overnight hospitalizations^b^	0.21	0	.19
Average number of visits to the emergency department^b^	0.36	0.36	.99

^a^Event was statistically significant.

^b^Two-week recall.

There were improvements in the averages of every health scale measured by the CHSA, with statistically significant improvements shown for the average physical health score of the child (from 65.64 preintervention to 76.28 postintervention, *P* = .045). In the two-week period prior to completing the final CHSA, as compared to the two-week period prior to completion of the baseline questionnaire, there was a significant decrease in the number of participants experiencing wheezing episodes (n = 9 to n = 2, *P* = .02), and a significant decrease in the average number of wheezing episodes per child (1.86 to 0.43, *P* = .02). There was also a decrease in the number of participants experiencing an asthma attack (n = 7 to n = 3) and a decreasing trend in the average number of asthma attacks (1.43 to 0.50), although these changes were not statistically significant. Our results also showed a significant decrease in the average number of visits to doctors’ offices or clinics due to asthma (1.23 to 0.38, *P* = .04). There were no overnight hospitalizations in the two-week period following the end of the pilot program, a nonsignificant reduction from an average of 0.21. In addition, prior to beginning this intervention, 5 children had never used a peak flow meter to help manage their asthma. After participating in this program, all children used a peak flow meter on a regular basis, and they also consistently reviewed their previous peak flow measurements with the program staff to see if their breathing had improved over time.

## Discussion

### Feasibility of a Computer-Assisted, School-Based, Intervention Program

This pilot project was intended to determine whether it was feasible to introduce a computer-based asthma self-management program in a low-income, urban, public elementary school. The pilot aimed to empower children’s understanding of their disease by teaching them to use a computer program to track their asthma symptoms and provide them with evidence-based feedback on their medication usage and symptom manifestations. The pilot demonstrated that a computer-based program, when used in a school-based setting, can lead to better self-management of asthma and thereby help reduce asthma exacerbations in children living in a low-income, urban setting. After participating in the pilot, there was improvement in wheezing symptoms, number of asthma episodes, and clinic visits. Our results showed the greatest significance when we paired baselines of preintervention participants with participants who completed a CHSA at the end of their second year of enrollment, suggesting that length of enrollment in the program increased the efficacy of the program. Thirteen of the 14 participants were enrolled in the program for 8 months or longer. These results suggest that students with more severe asthma tended to stay in the program longer. Those who stayed in the program for the longer period of time showed more significant improvements in outcomes when compared to those who stayed in the program for 7 months or less. These findings are consistent with other studies where participants with more severe asthma and enrolled for longer lengths of time demonstrated the greatest improvement in outcomes [24,41,42].

### Computer Programs as Tools for Asthma Self-management

The computer-based approach to asthma management has many advantages for intervening directly with children suffering from a chronic disease. This may be particularly true for lower-income populations. Low-income populations often lack regular physician supervision of their disease, leading them to receive care in emergency departments, usually when they are experiencing an acute asthma episode. One study of urban patients with asthma in 8 major US metropolitan areas found that over half of the study participants reported that it was difficult to get adequate care for an asthma attack or follow-up visits [11,43]. Another study found that children were more likely to use anti-inflammatory medications when they had seen a health care provider in the previous 6 months, and suggested that the discontinuity of care and the absence of follow-up care between physician visits may be factors contributing to the high rates of asthma in East Harlem [44]. By using the ALERTS computer program, participants’ health status is monitored on at least a weekly basis and this may help students adhere to prescribed medication plans, bridging the gap between intermittent care and the physician attention received only in situations of acute exacerbations.

Although it is not a substitute for the care and supervision by a physician, a program such as the one implemented here can help children and their families monitor medication usage and symptoms by providing personalized, real-time, evidence-based feedback on their disease status between physician visits. This information can be relayed to medically trained staff, either the school-based health center or the primary care physician, who can follow up with the patient and/or guardian if necessary. Additionally, the program generates weekly reports that are sent to the school-based health center and primary care physicians, providing a record of each patient’s disease and affording this population with better continuity of health care than they might otherwise receive. This can also alert health care providers to the children’s symptom history and use of medications.

Computers can be particularly effective in promoting knowledge of self-management skills among children with chronic diseases, such as asthma. Computers offer children an innovative method of monitoring symptoms that children find novel and fun. One study noted that children were more apt to use an interactive device than a written diary, for example [32]. Other studies have shown that electronic monitoring can clarify compliance among patients [36,45].

An important aspect of our pilot study was the school-based setting. Interventions similar to our pilot study have also utilized interactive technology, but were based in the home or in clinical settings, such as health clinics or doctors’ offices. Our program’s location within the students’ school overcomes some of the barriers other researchers have found in implementing large-scale asthma interventions in the home or in clinics. For example, some researchers have found that one of the greatest barriers to implementing an asthma education program in the home was the incompatibility of the schedules of families and nurse home visitors [46,47]. Families participating in interventions based in clinics might encounter some of the same scheduling difficulties. Children with asthma followed in the school setting would not be constrained by such family scheduling conflicts.

### Limitations

Limitations to our study included the fact that parents did not actually participate in any of the computer sessions (although they were involved in enrolling their children in the pilot program) and, therefore, might not have been as involved in their child’s asthma monitoring as those parents who partook of interventions based in the home or in clinics in other studies [48-50]. Another limiting factor is that children cannot benefit from the program on their own if they do not have access to a computer outside of the school sessions. Many low-income children do not have computers in the home; thus, school might be their only source of computer access. Importantly, the program is also available using an interactive voice response (IVR) platform (ie, telephone) in both English and Spanish that was not accessible in the school setting. Since people are more likely to have telephones than computers in their homes, this would increase the likelihood of being able to access and use the program. Indeed, in a parallel program to this one that was implemented in a group practice setting in Connecticut and used by patients at home, where both Internet and IVR access was available, 62% of program entries were made by IVR and 38% were Web-based [48,50]. Indeed, one of the authors (RJA) was the principal investigator of such a program implemented in three large pediatric practices in New York City that showed positive preliminary results [48,50].

### Conclusion

This pilot study demonstrated that an individualized, computer-assisted intervention resulted in improved health outcomes among low-income children in a public school setting. Increased awareness of the importance of regular peak flow meter measurements, provision of asthma trigger information, and a novel approach to symptom tracking resulted in fewer asthma exacerbations and improved overall physical health among a pediatric asthmatic population.

## Abbreviations

AIR: Asthma Intervention Report
ALERTS: Automated Live E-Health Response Tracking System
CHSA: Children’s Health Survey for Asthma
HIPAA: Health Insurance Portability and Accountability Act
IVR: interactive voice response
NHLBI: National Heart, Lung, and Blood Institute
PIN: personal identification number
